# Circumcision and Its Advantages

**Published:** 1910-08-20

**Authors:** Chas. A. Adair Dighton

**Affiliations:** Surgeon, Coburg Society Diseases of Women and Children; Assistant Surgeon, District Nursing Association, Cheltenham.


### THE GENERAL PRACTITIONER'S COLUMN.

[Contributions to this Column are invited, and, if accepted, will be paid for.]

### CIRCUMCISION AND ITS ADVANTAGES.

By CHAS. A. ADAIR DIGHTON, M.B., Ch.B.; Surgeon, Coburg Society Diseases of Women and Children; Assistant Surgeon, District Nursing Association, Cheltenham.

After reading an article on "Circumcision and its Abuses" in The Hospital, it seems to the writer only fair to give the other side of the question, and leave readers to judge for themselves. To start from a hygienic point of view, syphilis is a disease of great and increasing frequency in this country, yet very few cases are seen in the circumcised. It has been proved that syphilis can almost certainly be prevented by the use of ointment of mercury after copulation; this is easy in the circumcised, but almost impossible in the uncircumcised, as the folds of a prepuce, however small, are ideal for the growth of the spirochæta, and almost impossible to get at with mercury ointment. Then take gonorrhœa, said by Osler to be "one of the most widespread and serious of infectious diseases." In the circumcised there are no risks of balanitis, lymphangitis, and phagedæna. These advantages may be taken objection to from the moral point of view, but irregular intercourse has existed from the beginning of history, and will go on until the end, and with it the accompaniment of syphilis and gonorrhœa. It is impossible to stop the cause, and therefore surely any operation that is likely to alleviate the diseases is beneficial. I am sure if it were popularly known that circumcision, combined with mercury ointment after copulation, reduces the risk of syphilitic infection to a minimum, then circumcision would become general and syphilis a comparatively rare disease. In the article referred to the writer gives a long list of risks of the operation, none of which, in the course of much experience in this operation, I have seen. If it is done carefully under aseptic conditions it is a trivial operation, and if done within the first five days after birth, the risk of an anæsthetic is obviated, as it is quite unnecessary.

Of course, stretching of the prepuce, etc., is no doubt good, but does not possess the advantages of circumcision with regard to syphilis and gonorrhœa. Masturbation is far more common in the uncircumcised than the circumcised. Surely if this operation possibly prevents or alleviates such widespread diseases, such risks as mentioned in the "Abuse" article become merely details.

